# Correlation of the Radiographic Grading of Knee Osteoarthritis With Physical Function but Not Emotional Quality of Life Scores

**DOI:** 10.7759/cureus.75700

**Published:** 2024-12-14

**Authors:** Ramesh Radhakrishnan, Akshay Padki, Winston Shang Rong Lim, Daryl Zichen Cheng, Yeong Huei Ng, Kenny Xian Khing Tay, Joyce Suang Bee Koh, Tet-Sen Howe

**Affiliations:** 1 Orthopaedic Surgery, Singapore General Hospital, Singapore, SGP

**Keywords:** emotional outcomes, functional outcomes, joint, knee, osteoarthritis, physical outcomes, radiological scoring

## Abstract

Background

Multiple studies have shown that symptoms of knee osteoarthritis (OA) weakly correlate with the radiographic severity of the disease. Our objective was to determine possible correlations between the radiographic severity of knee OA and clinical manifestations such as disability, pain scores, and emotional health.

Methods

A retrospective review of registry data of 305 patients with knee OA was collected. The Kellgren-Lawrence and Ahlbäck classifications of radiographic knee OA were computed. These were correlated with the severity of functional limitations measured using the 36-Item Short-Form Health Survey (SF-36), Knee Society Score (KSS), and Oxford Knee Score (OKS). Statistical analysis was conducted with IBM SPSS Statistics for Windows, Version 22.0 (Released 2013; IBM Corp., Armonk, New York, United States). A p-value of 0.05 or less was considered statistically significant.

Results

There were no differences in BMI, gender, or operative site between all grades. There were significant differences in KSS Function scores between grade 2/3 patients and grade 4 patients. There were significant differences in OKS and SF-36 Physical Function between grade 2 and grade 4 patients. When comparing the loss of joint space with the functional scores, there were no statistically significant correlations.

Conclusion

Our study shows that increased radiological severity of knee OA was associated with increased limitation in the ability of patients to carry out their usual physical function. However, there was no significant correlation between radiological findings and non-tangible domains such as mental health, social functioning, and emotional role functions.

## Introduction

Knee osteoarthritis (OA) is a debilitating, functionally limiting disease process that is one of the leading causes of disability, affecting millions of people worldwide [1] and affecting 10% of men over the age of 60 and 13% of women [2]. Knee OA arises from aging, obesity, and joint trauma, with genetic predisposition and repetitive stress as key risk factors. Women, older adults, and individuals with prior knee injuries are more susceptible. The Framingham Osteoarthritis Study highlights obesity's role in joint stress and the cumulative effects of mechanical wear over time [1]. This multifactorial degenerative joint disease is characterized by progressive cartilage degradation, subchondral bone remodeling, and synovial inflammation. Enzymatic breakdown of the cartilage matrix, coupled with mechanical stress, reduces joint cushioning and leads to joint space narrowing. Synovitis amplifies inflammation through cytokine release, causing effusion, stiffness, and pain. Subchondral bone thickening and osteophyte formation further contribute to joint dysfunction. These pathological processes highlight OA's complex interplay of mechanical and inflammatory factors, driving pain and functional impairment [3-5].

Knee OA severely impairs quality of life and reduces mobility. Most OA patients suffer from great changes in their activities of daily living (ADLs), and approximately 25% of them have some kind of functional limitation, such as morning stiffness, reduced joint motion, crepitus, and muscle atrophy [6]. With a rapidly expanding elderly patient population, along with an emphasis on retaining mobility, quality of life, and the ability to continue working into their twilight years, knee OA is an increasingly significant cause of disease burden in the developed world [7].

Clinical symptoms such as pain and limited range of motion lead to functional impairments, loss of independence in daily tasks, depression, and social isolation for patients [6,8-11]. Nikolic et al. observed that patients tend to limit their joint use by avoiding physical activity, driven by discomfort, fear of pain, and the belief that increased activity might accelerate disease progression and cartilage loss, ultimately reducing their functional capacity [12]. The significant impairment in physical activities has a substantial impact on mental health, contributing to depression and a reduced quality of life, while also restricting daily tasks and self-care activities.

Knee OA is commonly diagnosed through physical examination, which assesses joint tenderness, swelling, range of motion, and crepitus, as well as plain orthogonal radiographs. Magnetic resonance imaging (MRI) offers detailed visualization of cartilage integrity, synovial inflammation, and subchondral bone changes, providing a more comprehensive understanding of structural abnormalities that may not be visible on radiographs but is not absolutely indicated for diagnosis. However, the plain radiographs that many rely on might not fully capture the patient's complete clinical condition. There is limited literature comparing radiographic knee OA severity with functional outcomes and subjective parameters such as emotional and mental health. Dowsey et al. have reported discrepancies in the correlation between the severity of radiographic features of knee OA and functional impairment [13]. Williams et al. found that self-reported disability in knee OA is closely related to concurrent pain and stiffness instead of radiological severity [14].

Various studies have tried but failed to identify a relationship between the multiple radiographic grading systems and the degree of pain felt by the patient [1,5,15]. Claessens et al. found that symptoms ranging from knee pain and swelling to deformity could not be predicted using standard anteroposterior (AP) and lateral knee radiographs [16]. With the exception of Lanyon et al. who found that osteophytes were the most closely associated radiographic feature with pain followed by loss of the tibiofemoral joint (TFJ) and patellofemoral joint (PFJ) spaces, respectively [17], the majority of current literature remains inconclusive regarding the correlation between radiographic findings of knee OA and pain scores [18-20].

The aim of this study was to analyze the correlation between objective radiographic severity of knee OA and patient-reported functional outcomes, pain scores, as well as emotional health.

## Materials and methods

Participants

This retrospective study of prospectively collected registry data was approved by the SingHealth Centralised Institutional Review Board (approval number: 2018/2300) and carried out per the ethical standards laid down in the 1964 Declaration of Helsinki. Informed consent was also obtained from all participants. Three hundred and five preoperative patients with knee OA who underwent total knee arthroplasty (TKA) in our tertiary institution (Singapore General Hospital, Singapore) by multiple, senior fellowship-trained orthopedic surgeons between January 2015 and December 2017 were selected. All the patients selected eventually went on to have TKA performed and were followed up for a minimum of two years. Patients who suffered from post-traumatic arthritis, underwent bilateral TKA, or had undergone a contralateral TKA were excluded from this study as were patients who were under the age of 60.

Radiographic and patient evaluation

Preoperative three-view weight-bearing radiographs of the affected knee were selected to capture the period of peak symptom severity. Two trained physicians independently calculated radiographic measurements, including medial, lateral, and patellofemoral joint space in millimeters. These measurements were then used to grade the severity of OA using the Kellgren-Lawrence (KL) and Ahlbäck classification systems [17-19].

The KL grading of OA severity ranges from grade 0 measured as no radiographic features of OA to grade 4, large osteophytes, marked joint space narrowing, severe sclerosis, and definite bony deformity (Table 1). For a more objective measure, the Ahlbäck classification was used ranging from grade 1 showing joint space narrowing of less than 3 mm to grade 5 showing severe bone attrition of more than 10 mm (Table 2). 

**Table 1:** The Kellgren-Lawrence classification

Kellgren-Lawrence classification	Description
Grade 0	Definite absence of X-ray changes of osteoarthritis
Grade 1	Doubtful joint space narrowing and possible osteophytic lipping
Grade 2	Definite osteophytes and possible joint space narrowing
Grade 3	Moderate multiple osteophytes, definite narrowing of joint space, some sclerosis, and possible deformity of bone ends
Grade 4	Large osteophytes, marked narrowing of joint space, severe sclerosis, and definite deformity of bone ends

**Table 2:** The Ahlbäck classification

Ahlbäck classification	Description
Grade 1	Joint space narrowing (<3 mm)
Grade 2	Joint space obliteration
Grade 3	Minor bone attrition (0-5 mm)
Grade 4	Moderate bone attrition (5-10 mm)
Grade 5	Severe bone attrition (>10 mm)

In the final grading system, a combination of KL and Ahlbäck was collated to form an aggregate score which was then graded 1-4 based on severity (Table 3). All measurements taken and calculated were validated for accuracy with minimal inter-observer variability noted. 

**Table 3:** Derivation of the final grading used in current study based on the Kellgren-Lawrence and Ahlbäck radiographical grades

Final grade	Kellgren-Lawrence+Ahlbäck grades
Grade 1	1-3
Grade 2	4-5
Grade 3	6-7
Grade 4	8-9

**Figure 1:**
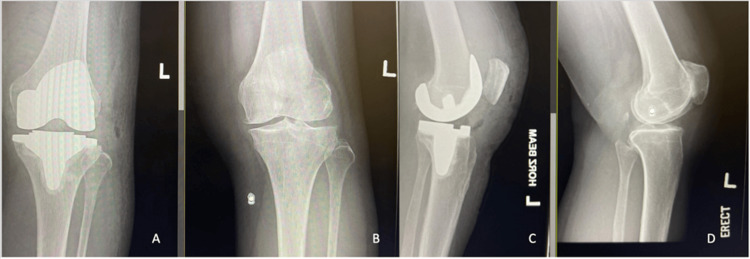
Preoperative (B and D) and postoperative (A and C) X-ray images of a patient with moderate osteoarthritis

**Figure 2:**
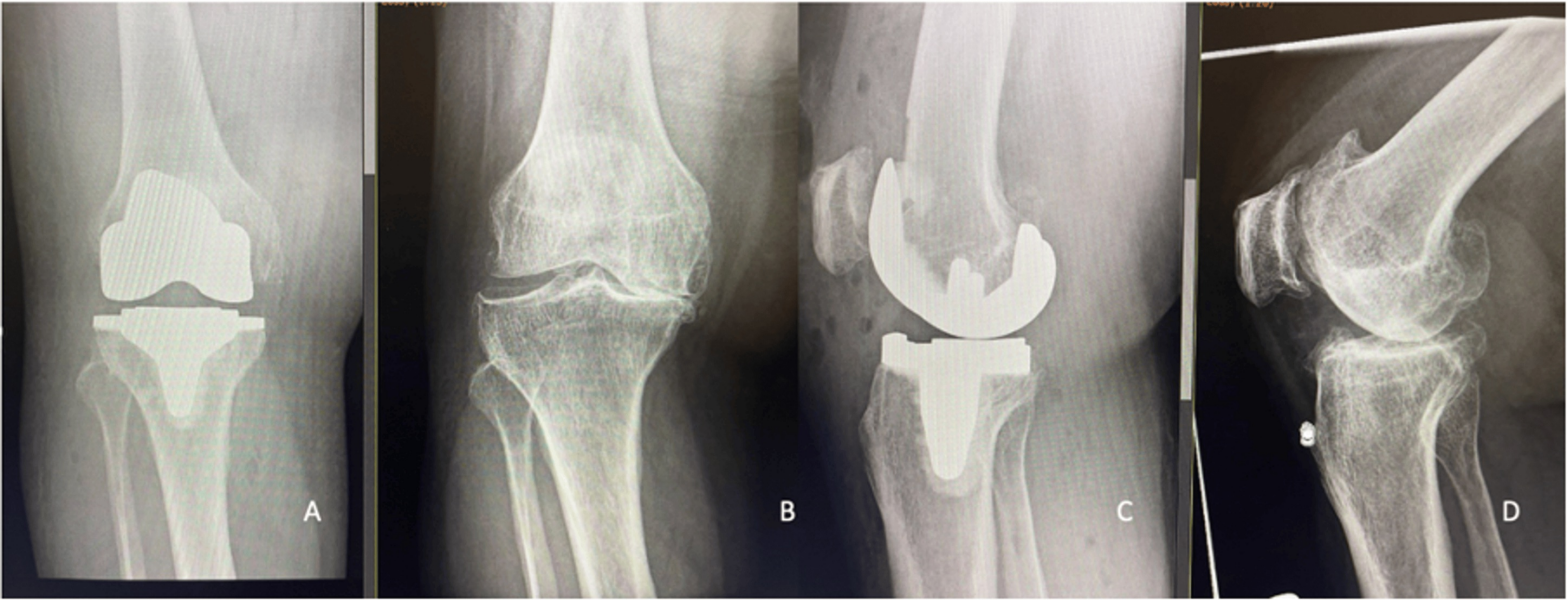
Preoperative (B and D) and postoperative (A and C) X-ray images of a patient with severe osteoarthritis

Figure 1 and Figure 2 show the preoperative and postoperative X-ray images of study subjects with moderate and severe knee OA who had undergone TKA.

The primary clinical outcomes were assessed using the 36-Item Short-Form Health Survey (SF-36), Knee Society Score (KSS), and Oxford Knee Score (OKS). Clinical outcome scores were measured preoperatively by independent, trained physiotherapists involved in the postoperative evaluation of all orthopedic patients at our institution, within four weeks prior to their respective operative dates. The OKS is a 12-point questionnaire that measures the patient's pain and disability [20]. The score ranges from a minimum of 12 points to a maximum of 60 points with a lower score indicating greater severity. The SF-36 is a health questionnaire containing 36 questions across eight health domains. These include physical functioning, role limitations due to health issues (role functioning physical), pain, general health, mental health, role limitations due to emotional issues (role functioning emotional), social functioning, and vitality. Each domain is allocated a total of 100 points with a higher score indicating better functional outcomes. The KSS consists of the Knee Society Knee score as well as the Knee Society Function score. In KSS, higher scores indicated greater severity of disease. Both the SF-36 and OKS have been validated for use in Singapore [2].

Electronic medical records were also reviewed, and baseline patient sociodemographic characteristics including, age, sex, ethnicity, BMI, and site of surgery were also collected.

Statistics

Statistical analysis was conducted with IBM SPSS Statistics for Windows, Version 22.0 (Released 2013; IBM Corp., Armonk, New York, United States). Demographic characteristics and functional assessments were evaluated using a one-way ANOVA test. If the one-way ANOVA showed statistical significance, a post hoc analysis with Tukey's honestly significant difference (HSD) and effect size calculation (partial eta-squared (η²)) was conducted. Statistical significance was defined as a p-value of less than 0.05.

## Results

Of the 305 patients selected for this study, 219 were women compared to 86 men. All patients were receiving their first unilateral TKA with 143 right knees compared to 162 left. The median age was 67.3±9.4. Preoperative patient demographics are shown in Table 4.

**Table 4:** One-way ANOVA test analysis of preoperative patient demographics

	Grade 1 (n=13)	Grade 2 (n=82)	Grade 3 (n=117)	Grade 4 (n=93)	P-value
Age	64.2±8.4	64.7±7.2	67.1±7.4	68.7±7.5	0.003
Gender					
Female	7	57	85	71	0.351
Male	6	25	32	22	
BMI	26.5±4.2	27.5±5.2	28.0±4.5	28.4±5.1	0.418
Op site					
Left	7	38	59	39	0.622
Right	6	44	58	54	

The distribution of patients across grades 1-4 was 13, 82, 117, and 93, respectively. There was strong inter-rater reliability for both the KL (ICC: 0.82; 95% CI: 0.68-0.89) and Ahlbäck (ICC: 0.87; 95% CI: 0.77-0.92) radiographic classifications (p<0.001). Patients with grade 2 OA were significantly younger than those with grade 4 (post hoc p=0.003). No significant differences in age were observed between the other grades, and there were no notable differences in BMI, gender, or operative site across the grades.

There was a statistically significant decline in KSS Function scores between patients in grade 2 and grade 3 (post hoc p=0.017), as well as between grade 2 and grade 4 (post hoc p<0.001). Additionally, significant differences in KSS Knee scores were observed between grade 1 and grade 4 patients (Table 5) (post hoc p=0.016).

**Table 5:** One-way ANOVA test analysis of the comparison of radiological severity with preoperative function and quality of life KSS: Knee Society Score; OKS: Oxford Knee Score; SF-36: 36-Item Short-Form Health Survey

	Grade 1 (n=13)	Grade 2 (n=82)	Grade 3 (n=117)	Grade 4 (n=93)	P-value
KSS Function	50.8±24.3	58.0±15.3	50.8±16.1	47.7±18.4	0.001
KSS Knee	42.0±16.8	37.9±14.4	32.7±14.9	29.1±13.9	0.000
OKS	38.2±9.2	33.9±7.4	36.8±7.7	36.8±7.7	0.018
SF-36 Physical Function	26.9±40.1	19.1±33.8	15.8±30.5	15.2±31.0	0.001
SF-36 Role Physical	31.8±41.9	20.7±34.6	15.9±31.2	14.1±30.0	0.562
SF-36 Bodily Pain	35.2±17.0	33.4±17.4	33.1±16.9	30.8±14.9	0.640
SF-36 General Health	62.2±24.5	68.0±20.1	68.3±21.1	66.6±20.4	0.474
SF-36 Vitality	51.9±18.3	69.4±20.8	69.7±21.1	69.3±22.7	0.040
SF-36 Social Function	51.9±29.7	58.6±36.3	52.6±34.7	47.9±34.5	0.257
SF-36 Role Emotional	97.4±9.2	88.9±31.6	90.0±29.4	88.0±31.5	0.757
SF-36 Emotional Health	75.1±16.9	80.2±17.2	82.7±15.7	81.1±14.9	0.351

Significant differences were observed in the OKS (post hoc p=0.026) and the SF-36 Physical Function score (post hoc p<0.001) when comparing grade 2 patients to those with grade 4 (Table 6). Of interest, there was no significant correlation between radiological severity gradings and SF-36 outcomes of pain, social outcome, emotional health, and limitations in usual role activities due to physical or emotional problems. The effect size η² for KSS Function, KSS Knee, and OKS was 0.05, 0.06, and 0.33 respectively. 

**Table 6:** Pearson's correlation coefficient between the various radiographic scoring systems and preoperative function KSS: Knee Society Score; OKS: Oxford Knee Score; SF-36: 36-Item Short-Form Health Survey

	Kellgren-Lawrence	P	Ahlbäck	p
KSS Function	-0.190	0.001	-0.199	0.0004
KSS Knee	-0.251	0.000009	-0.221	0.000096
OKS	0.113	0.050	0.105	0.068
SF-36 Physical Function	-0.185	0.001	-0.165	0.004

In the comparison of joint space loss and functional scores, a significant correlation was found between medial joint space and deteriorating KSS, with a correlation coefficient of 0.233. Of notable interest, this study found that medial joint space narrowing most closely correlated with the severity of functional disability as determined by OKS and KSS Knee and Function scores (Table 7).

**Table 7:** Pearson's correlation coefficient between joint line thickness on radiographs and preoperative function KSS: Knee Society Score; OKS: Oxford Knee Score; SF-36: 36-Item Short-Form Health Survey

	Medial joint thickness	P	Lateral joint thickness	p
KSS Function	0.129	0.025	0.016	0.781
KSS Knee	0.233	0.000043	-0.106	0.066
OKS	-0.117	0.042	-0.06	0.274
SF-36 Physical Function	0.167	0.004	0.015	0.795

## Discussion

Our study found that the radiological severity of knee OA negatively correlated with patient-reported physical function scores. However, there was no significant correlation between pain scores and emotional health.

Kocak et al. found that patients with higher radiological grades of knee OA were associated with poor functional ability. They found patients with KL grade 4 had significantly reduced quadriceps/knee extensor strength, range of motion, and functional scores compared to patients under KL grade 3 [21]. In a population-based study involving 819 adults experiencing knee pain, Duncan et al. reported a positive correlation between the radiological severity of knee OA and both stiffness and a decline in physical function [22]. A direct positive relationship has been similarly described between radiographic features of knee OA and functional disability in existing literature [12,23-27]. These are consistent with our findings of poorer physical function scores in association with higher radiological severity of knee OA.

Interestingly, our study found that grade 1 patients had similar functional outcomes to those with higher grades. We hypothesize that given all patients recruited into this study went on to eventually have TKA, it can be argued that all patients in this cohort had severe functional impairment. Radiological findings in patients with knee OA may not always be associated with functional capacity [28]. This subset of patients likely had pain generators that were not evident on plain radiographs including early inflammatory mediators in knee OA or were suffering from referred pain from other anatomical regions. This finding aligns with the research by Roemer et al., which proposed that OA is not solely a cartilage disease but affects the entire joint, including surrounding soft tissues, ultimately contributing to joint failure [29].

Pain is the main complaint among patients with knee OA, but there is existing discordance in using the radiographic severity of knee OA when assessing for pain in the knee in several current studies [17-19,30-33]. Findings of the present study support this stance with no correlation found between the SF-36 Bodily Pain domain and the radiological severity of knee OA.

While the current body of literature supports the theory that knee OA carries a significant disease burden with a significant mental, societal, and emotional toll [7,34,35], scores of the SF-36 Emotional Health domain showed no correlation to the radiological severity of knee OA. This seeming paradox could be attributed to a cause versus effect, i.e., poor mental health may result in poorer coping mechanisms, rather than the severity of knee OA, as assessed radiologically, leading to poorer mental health. Poor mental health has been shown to be an important predictor of physical functioning in the healthy elderly and those with knee OA [36,37]. There was also no significant correlation between the SF-36 Social Function domain score and the SF-36 Physical Function domain score with the radiological severity of knee OA, and similar findings were also reported by Steenkamp et al. [31].

Although Bruyere et al. found that medial joint space narrowing was not associated with an increase in pain [24], our study showed that it correlated most closely with worsening physical function scores as measured on KSS. We hypothesize that pain alone may not be the main determinant of disability. Rather, the attendant loss of coronal alignment and the possible attendant loss of knee motion translate more to disability in common ADLs such as stair climbing, squatting, and sitting on lower stools.

When comparing the two grading systems, the KL grading system was able to better predict physical, mental, and emotional disability. One possibility could be because the KL grading system incorporated not only joint space narrowing but also the presence of osteophytes, which concurs with Lanyon et al. who found that osteophytes were the most closely associated radiographic feature with pain [17]. Özden et al. also discovered a strong correlation between the KL grade and both the Visual Analog Scale (VAS) for pain and the patient's level of activity [38].

Our results emphasize the necessity of conducting a comprehensive clinical assessment of patients to evaluate the overall severity of knee OA. Decisions regarding surgical intervention should not rely exclusively on the radiographic severity of knee OA. When advising patients with advanced knee OA who might qualify for knee arthroplasty, it is crucial to take into account their pain levels and self-reported limitations in daily activities rather than depending solely on standard radiographs. Artificial intelligence (AI)-guided systems can improve the ratings of knee radiographs and show a stronger association with clinical severity. With the advent of AI, machine learning, and computer-assisted diagnostics, fine-tuning the radiographic grading of knee OA for better prognostication will allow clinicians to better advise patients when considering surgical intervention. AI systems for diagnosing knee OA include deep learning models for radiographic analysis, machine learning algorithms integrating clinical and imaging data, natural language processing tools for electronic health record extraction, and AI-powered MRI platforms detecting cartilage degeneration and joint abnormalities, enhancing early detection and personalized treatment approaches [39]. Neubauer et al. found that AI-aided diagnostic ratings had a higher association with the overall KL score and clinical outcome score compared to AI-unaided ratings [40].

Strength and limitations

One notable strength of our study is that our registry data is robust with extended follow-up. One limitation of this study was in the patient selection, as all patients recruited into this study went on to eventually have TKA, it can be argued that all patients in this cohort had significant functional impairment. Another limitation is the absence of data regarding potential confounders, including smoking, physical activity, analgesic use, and comorbidities.

## Conclusions

This study demonstrated that the radiological severity of knee OA, as assessed through a combination of scoring systems, correlated with declining functional scores. However, the emotional health of patients with knee OA did not show a correlation with the severity of radiological findings. Hence, when advising patients with advanced knee OA about the possibility of knee arthroplasty, it is crucial to consider their pain levels and self-reported difficulties in daily activities, rather than relying only on the severity shown in radiographs.
